# In memoriam: George L. Drusano

**DOI:** 10.1128/aac.01045-25

**Published:** 2025-08-19

**Authors:** Cesar A. Arias

**Affiliations:** 1 Division of Infectious Diseases, Houston Methodist Hospital, Houston, Texas, USA; 2 Center for Infectious Diseases, Houston Methodist Research Institute 167626 , Houston, Texas, USA; 3 Department of Medicine, Weill Cornell Medical College 12295 , New York, New York, USA

## EDITORIAL

On June 10, 2025, the scientific community lost George L. Drusano, MD, of the University of Florida. George will always be remembered not only for his pioneering work on antimicrobial pharmacokinetics and pharmacodynamics and his contributions to our esteemed journal Antimicrobial Agents and Chemotherapy but also for his curiosity, sense of humor, and dedication to translational science to make lives better. George generated pioneering data that provided critical insights into the manner in which antibiotics kill and prevent resistance using pharmacological approaches. Our journal will be forever indebted to his contributions.

**Figure:**
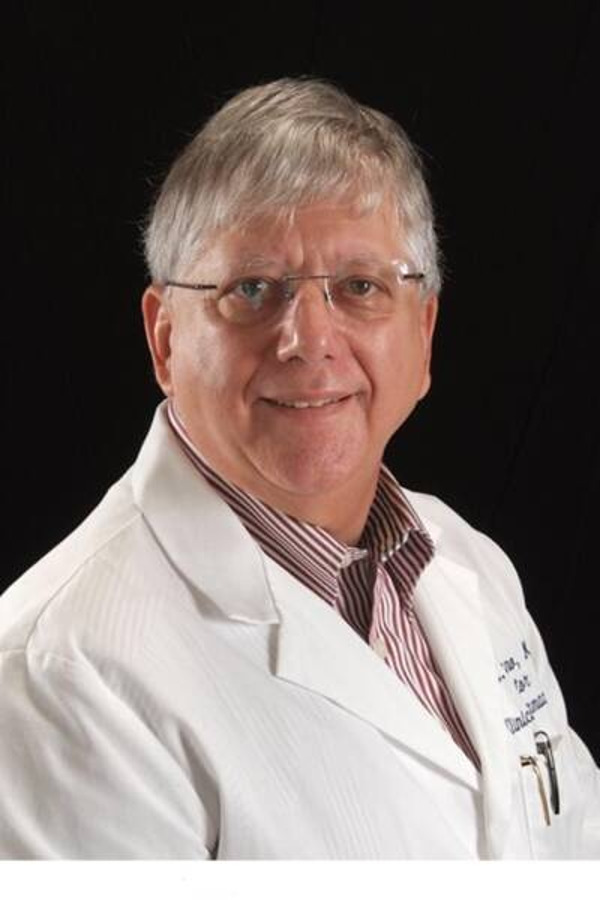


## IN MEMORIAM

On June 10, 2025, George L. Drusano, MD, died at the age of 76 years. George leaves behind his beloved wife, Marianne, and three sons, George Junior (Chip), Michael, and Stephen, and his son-in-law, Chad. George, who was larger than life, was a visionary leader, scientist, physician, friend, and mentor. His pioneering work in antimicrobial pharmacokinetics and pharmacodynamics (PK/PD) laid the foundation for modern anti-infective therapy. But to those who knew him, George was far more than a brilliant researcher. He was an unapologetically curious thinker, a generous collaborator, and a fiercely committed advocate for antimicrobial PK/PD. With an unwavering dedication to translational medicine, he devoted his life to bridging the gap between mechanistic science and patient care and ensuring that this knowledge could be used to save lives. George developed and refined PK/PD modeling strategies that have become central to antimicrobial dosing and resistance suppression, spanning nearly every major therapeutic class, including β-lactams, fluoroquinolones, aminoglycosides, and oxazolidinones. His work extended across bacteriology, mycobacteriology, and virology, including those of biodefense concern, and always with the same goal: to transform insight into impact and ensure patients received optimal dosing regimens.

One of George’s most profound contributions was his deep and sustained exploration of the complex interplay among drug, pathogen, and host, and how these relationships could be leveraged to improve outcomes in patients with drug-resistant infections. Through his use of *in vitro* hollow-fiber and *in vivo* neutropenic murine thigh and lung infection models, George generated critical insights into antimicrobial killing dynamics, exposure-response relationships, and the suppression of resistance. These platforms became foundational tools in the development of modern antimicrobial regimens, informing dosing strategies used in clinical practice and shaping regulatory approvals worldwide. His mastery of combination therapy and resistance suppression redefined treatment paradigms and helped elevate pharmacometrics from a niche academic pursuit into a central pillar of clinical infectious diseases. Today, the principles George helped establish will serve as the basis for emerging approaches that integrate artificial intelligence and machine learning into individualized therapeutic drug dosing. This is a testament to the vision and durability of his work.

Among his many innovations, George was the first to introduce the concept of stochastic forecasting, known as Monte Carlo simulation, to antimicrobial regimen design. This approach, now a mainstay in dose selection and drug development, changed how the field evaluates drug exposures to optimize efficacy. His influence was especially important with regard to guiding therapy for difficult-to-treat infections caused by *Pseudomonas aeruginosa*, *Acinetobacter baumannii*, carbapenem-resistant Enterobacterales, multidrug-resistant tuberculosis, and fluoroquinolone-resistant *Bacillus anthracis*. His insights led to optimized regimens for life-threatening conditions, such as pneumonia and bloodstream infections, where precision in therapy can be the deciding factor between life and death.

George’s scientific output was extraordinary. He authored more than 300 peer-reviewed articles, including landmark papers in *Antimicrobial Agents and Chemotherapy*, *Clinical Infectious Diseases*, *The Journal of Infectious Diseases*, and *The Lancet Infectious Diseases*. His work has been cited nearly 30,000 times, reflecting its broad and lasting influence. His research was continuously supported by government, industry, and private foundations, including multiple R01 and P01 awards from the NIH. Importantly, his research was never just academic; it directly informed clinical practice, shaped public health strategies, and improved patient outcomes around the world.

George also gave generously of his time and expertise in service to the broader infectious diseases and pharmacology communities. He held numerous leadership roles, including President of the International Society for Anti-Infective Pharmacology (ISAP), and served on the IDSA Program Committee, the Interscience Conference on Antimicrobial Agents and Chemotherapy (ICAAC) Program Committee, and multiple NIH/NIAID review panels. He was an *ad hoc* member of the NIAID Council, where he advised on emerging challenges in bacterial resistance and biodefense and consulted for both NIAID and the CDC. George also contributed to global antimicrobial resistance (AMR) efforts through advisory roles at the U.S. FDA, EMA, WHO, and NIH. He spent a decade as editor of the pharmacology and experimental therapeutics section of *Antimicrobial Agents and Chemotherapy* and also contributed his expertise to *mBio* and several other leading journals.

His accomplishments were recognized through numerous awards, including the Paul Ehrlich “Magic Bullet.” Award (2015), the Cubist-ICAAC Award from the American Society for Microbiology (2013), the Maxwell Finland Award for Scientific Achievement from the National Foundation for Infectious Diseases (2012), and the Distinguished Investigator of the Year award from the American College of Clinical Pharmacology (2003). He held academic appointments at the University of Maryland, Albany Medical College, and most recently, the Institute for Therapeutic Innovation at the University of Florida, where he was honored with a UF Research Foundation Professorship. George was a Fellow of both the Infectious Diseases Society of America and the American Academy of Microbiology, and a member of the American Federation for Clinical Research.

What truly set George apart was how deeply he cared for the science, for the people doing the work, and for the patients it served. He had an extraordinary gift for making complicated ideas clear and manageable, and he did it with an energy that made you want to rise to the occasion. George expected a lot of his colleagues. He was sharp, probing, always ready to challenge your thinking, but he gave even more in return. He never hesitated to take the extra time to walk you through his thinking. If you did not immediately grasp one of his mathematical concepts (something that was second nature to him given he was a physics major), he would patiently break it down until you did. Above all, George was most proud of the fellows and mentees he trained; he saw their growth and success as his greatest legacy. His mentees and colleagues in both academic medicine and the pharmaceutical industry describe his mentorship as transformative, his scientific standards as unwavering, and his personal kindness as enduring. We all quickly learned to listen closely to what George said so that we would not miss the point, which was often a window into something profoundly thoughtful. Regarding the efforts of others, George made you feel that the work mattered, and that you mattered, too. That same sentiment extended to his patients. In the earliest days of the HIV-AIDS epidemic, when fear and stigma kept many physicians away, George stepped forward. With great compassion, he treated and stood by patients even when there were no effective therapies and when the outcomes were heartbreakingly inevitable. That was George through and through; someone who showed up, especially when it was hard. He never wavered when people needed him most.

George’s legacy is not just in the papers he wrote, the models he built, or the policies he shaped but the lives he changed and the field he helped define. Beyond the science, George was a man of faith who loved his family. He was known for his infectious energy and unforgettable humor. His presence would light up a room, whether through a perfectly timed movie reference, a John Wayne impression, or a dry one-liner that left everyone laughing. George loved to challenge both ideas and people, often wrapping deep insight and sharp intellect in a layer of humor that was uniquely his. He had a remarkable way of making people feel valued and seen, even in the middle of the most intense scientific discussions. When asked what motivated him each morning to continue his life’s work, he referenced the 1980s TV series The A-Team, saying, “I do it for the jazz.” By this, he meant there is nothing more fulfilling than dedicating himself to work that can ultimately be transformed into life-saving therapies for patients with serious infections. Even with this undeniable calling, George reminded us that it was not just about the science, but the connections forged among one another. And that may be the most enduring lesson he left behind.

George often reflected on the evolution of PK/PD, lamenting how much of its foundational knowledge had been lost following the era of Harry Eagle. He feared this might happen again and made it his mission to ensure that the field would not fade into obscurity. That responsibility now rests with all of us, his colleagues, mentees, and friends, to ensure that his legacy endures, that the science he helped create continues to thrive, and that the underlying fidelity to the field that he demonstrated remains central to how we care for patients. May we honor his memory by advancing the science he championed and ensuring that the principles of PK/PD continue to guide how we treat infections and protect lives for generations to come.

George, in your own immortal words, you made “like a hockey player and got the puck out of here.” We mourn your loss and miss you more than these words can convey. You have changed our lives for the better. Rest in peace, our dear friend.

This tribute was written by the following:

Thomas P. Lodise, Albany College of Pharmacy and Health Sciences, Albany, New York, USA

Sujata M. Bhavnani, Institute for Clinical Pharmacodynamics, Inc., Schenectady, New York, USA

Paul G. Ambrose, Institute for Clinical Pharmacodynamics, Inc., Schenectady, New York, USA

Marc H. Scheetz, Department of Pharmacy Practice, College of Pharmacy, Midwestern University, Downers Grove, Illinois, USA, Pharmacometrics Center of Excellence, Midwestern University, Downers Grove, Illinois, USA, Department of Pharmacy, Northwestern Memorial Hospital, Chicago, Illinois, USA and Department of Pharmacology and Biomedical Sciences, College of Graduate Studies, Midwestern University, Downers Grove, Illinois, USA

Charles (Chuck) A. Peloquin, Department of Pharmacy, UF Health Shands Hospital, Gainesville, FL 32608, USA and Division of Infectious Diseases, College of Medicine, University of Florida, Gainesville, FL 32610, USA

Michael N. Neely, Keck School of Medicine, University of Southern California, Los Angeles, California, USA, Laboratory of Applied Pharmacokinetics and Bioinformatics, The Saban Research Institute, Children's Hospital of Los Angeles, Los Angeles, California, USA and University of Pittsburgh, School of Medicine, Pittsburgh, Pennsylvania, USA

Vincent H. Tam, Department of Pharmacological and Pharmaceutical Sciences, University of Houston College of Pharmacy; Houston, Texas, 77204, United States of America and Department of Pharmacy Practice and Translational Research, University of Houston College of Pharmacy; Houston, Texas, 77204, United States of America

Robert A. Bonomo, Research Service, Louis Stokes Cleveland Department of Veterans Affairs, Cleveland, OH 44106, Department of Biochemistry, Case Western Reserve University, Cleveland, OH 44106, Department of Medicine, Case Western Reserve University, Cleveland, OH 44106, Department of Molecular Biology and Microbiology, Case Western Reserve University, Cleveland, OH 44106, Department of Pharmacology, Case Western Reserve University, Cleveland, OH 44106, Department of Proteomics and Bioinformatics, Case Western Reserve University, Cleveland, OH 44106, VA Center for Antimicrobial Resistance and Epidemiology, Case Western Reserve University, Cleveland, OH 44106, Case Western Reserve University School of Medicine, Cleveland, Ohio, USA, Louis Stokes Cleveland VA Medical Center, Cleveland, Ohio, USA

John S. Bradley, University of California, San Diego, California, USA and Rady Children’s Hospital, San Diego, California, USA

Ashley N. Brown, Institute for Therapeutic Innovation, Department of Medicine, College of Medicine, University of Florida, Orlando, FL 32827, USA and

Department of Pharmaceutics, College of Pharmacy, University of Florida, Orlando, FL 32827, USA

Dennis M. Dixon, National Institutes of Health, Retired

Michael N. Dudley, Qpex Biopharma, Inc., San Diego, California, USA

Henry(Hank) S. Heine, Institute for Therapeutic Innovation, University of Florida, Orlando, Florida, USA

William Hope, The University of Liverpool, Liverpool, UK

Arnold Louie, Institute for Therapeutic Innovation, University of Florida, Orlando, FL, USA

Manjunath (Amit) P. Pai, Department of Clinical Pharmacy, College of Pharmacy, University of Michigan, 428 Church St, Ann Arbor, MI, 48108, USA, amitpai@umich.edu

Bret K. Purcell, Institute for Therapeutic Innovation, University of Florida, Orlando, Florida, USA

Keith A. Rodvold, University of Illinois Chicago, Chicago, Illinois, USA

Fritz Sorgel, IBMP - Institute for Biomedical and Pharmaceutical Research, 90562 Nürnberg-Heroldsberg, Germany, Institute of Pharmacology, West German Heart and Vascular Center, University of Duisburg - Essen, Germany

Chad Testa, Curza, Salt Lake City, Utah, USA

